# FoxO gene family evolution in vertebrates

**DOI:** 10.1186/1471-2148-9-222

**Published:** 2009-09-07

**Authors:** Minghui Wang, Xiangzhe Zhang, Hongbo Zhao, Qishan Wang, Yuchun Pan

**Affiliations:** 1 School of Agriculture and Biology, Department of Animal Sciences, Shanghai Jiao Tong University, Shanghai, 200240, PR China; 2 Shanghai Key Laboratory of Veterinary Biotechnology, Shanghai 200240, PR China

## Abstract

**Background:**

Forkhead box, class O (FoxO) belongs to the large family of forkhead transcription factors that are characterized by a conserved forkhead box DNA-binding domain. To date, the FoxO group has four mammalian members: FoxO1, FoxO3a, FoxO4 and FoxO6, which are orthologs of DAF16, an insulin-responsive transcription factor involved in regulating longevity of worms and flies. The degree of homology between these four members is high, especially in the forkhead domain, which contains the DNA-binding interface. Yet, mouse FoxO knockouts have revealed that each FoxO gene has its unique role in the physiological process. Whether the functional divergences are primarily due to adaptive selection pressure or relaxed selective constraint remains an open question. As such, this study aims to address the evolutionary mode of FoxO, which may lead to the functional divergence.

**Results:**

Sequence similarity searches have performed in genome and scaffold data to identify homologues of FoxO in vertebrates. Phylogenetic analysis was used to characterize the family evolutionary history by identifying two duplications early in vertebrate evolution. To determine the mode of evolution in vertebrates, we performed a rigorous statistical analysis with FoxO gene sequences, including relative rate ratio tests, branch-specific *d*_*N*_/*d*_*S *_ratio tests, site-specific *d*_*N*_/*d*_*S *_ratio tests, branch-site *d*_*N*_/*d*_*S *_ratio tests and clade level amino acid conservation/variation patterns analysis. Our results suggest that FoxO is constrained by strong purifying selection except four sites in FoxO6, which have undergone positive Darwinian selection. The functional divergence in this family is best explained by either relaxed purifying selection or positive selection.

**Conclusion:**

We present a phylogeny describing the evolutionary history of the FoxO gene family and show that the genes have evolved through duplications followed by purifying selection except for four sites in FoxO6 fixed by positive selection lie mostly within the non-conserved optimal PKB motif in the C-terminal part. Relaxed selection may play important roles in the process of functional differentiation evolved through gene duplications as well.

## Background

Mammalian FoxO proteins (FoxO1, FoxO3a, FoxO4 and FoxO6) which are homologous to Caenorhabditis elegans protein DAF-16, belong to the O ('other') class of the Fox superfamily [1,2]. FOXO1 is the first identified member of the FoxO family of transcription factors [3] and is involved in the transcriptional activity of alveolar rhabdomyosarcomas [3]. Since then, the discovery of mammalian FoxO genes has grown rapidly, now FoxO proteins have been identified in several different organisms, including zebrafish, mouse, rat and human. As transcription factors in the nucleus, the primary function of FoxO proteins is to bind to their cognate DNA targeting sequences as monomers. The co-crystal structure of HNF-3γ with DNA shows that there are 14 protein-DNA contacts distributing throughout the forkhead domain, but the third α-helix (H3) plays the most important role in a winged helix/forkhead protein's DNA-binding specificity [4]. In addition, both winged loops also make important interactions with DNA [4,5]. Although the molecular basis of the DNA-binding specificity of FoxO transcription factors is poorly understood, high-affinity DNA-binding studies have identified a consensus FoxO-recognized element (FRE) as (G/C) (T/A)AA(C/T)AA [6-8]. Indeed, functional FRE sites that match this consensus sequence have been identified in the promoters of many genes, such as Fas ligand (FasL), insulin-like growth factor binding protein 1 (IGFBP1) and the apoptotic regulator BIM[9,10]. Additional putative FoxO-target genes and their potential cis-regulatory binding sites have been predicted by systematic bioinformatic approaches [11]. Thus, FoxO transcription factors appear to be involved in various signaling pathways and control a wide range of biochemical processes including cellular differentiation, tumor suppression, metabolism, cell-cycle arrest, cell death, and protection from stress [1,9,10]. In the mouse, four different FoxO members have been identified to date: Foxo1, Foxo3, Foxo4 and Foxo6 [12,13]. FoxO6 is the latest member of the FoxO family to be cloned and shares significant sequence similarity with the other members of the family [13].

FoxO1 and its close paralogus (FoxO3, FoxO4 and FoxO6) are thought to some degree of functional diversification during development [14-16] and their potential physiological roles might be different [14]. Indeed, a rapid overview of the data collected on FoxO1, 3, 4 and 6 highlights how these proteins may be different. First, each FoxO gene showed different expression patterns in tissues [7,12,17-19]. While Foxo1 was strongly expressed in the striatum and neuronal subsets of the hippocampus (dentate gyrus and the ventral/posterior part of the CA regions), Foxo3 was more diffusely expressed throughout the brain including all hippocampal areas, cortex and cerebellum, and Foxo6 expression was eminent in various parts of the adult mouse brain. Moreover, the individual disruption of Foxo1, Foxo3 and Foxo4 genes in mice results in different phenotypes [14,16]. While a homozygous knockout of Foxo1 (FKHR) was embryonic lethal due to failures in angiogenesis and vessel formation, Foxo3a-/- (FKHRL1) and Foxo4-/- (AFX) were viable and appeared to develop normally. Later in development, Foxo3a-/- females were found to be age-dependently infertile and showed abnormal ovarian follicular development. As to the physiological role, each FoxO gene exhibits a distinct response under a variety of conditions [20-22]. Therefore, despite the high sequence identity shared by FoxO genes domain (more then 60% in humans [17]), the physiological roles of FoxO genes are functionally diverse in mammals.

Single copy genes are thought to evolve conservatively because of strong negative selective pressure. Gene duplications produce a redundant gene copy and thus release one or both copies from negative selection pressure [23]. There are a number of models for the fate of duplicate gene that predict functional differentiation of paralogs based on protein sequence or regulatory divergence [24,25]. Currently four most prominent models are neofunctionalization [26], subfunctionalization [24], the Dykhuizen-Hartl effect [27] and adaptive diversification. Very recently, the list has been expanded by the introduction of the subneofunctionalization [28] and the adaptive radiation [29] models that predict rapid subfunctionalization after duplication followed by a prolonged period of neofunctionalization and adaptive divergence of duplicate genes in a process analogous to species radiations, respectively. Thus, duplications are thought to be an important precursor of functional divergence [30]. Here, we are interested in the specific role that natural selection might play in the evolutionary history of this gene duplication.

The increased availability of FoxO sequences in the public databases allows us to explore the functional diversity from a phylogenetic perspective within the FoxO family in vertebrates. The study was conducted by analyzing amino acid and nucleotide-based divergence data from different species covering the entire vertebrates. Our aim was to elucidate the evolutionary mechanisms operating in the retention of these genes and evaluate the changes in selection pressures following duplication. We also identified the sites under positive Darwinian selection. Finally, we tried to map the positively selected sites to the structural and functional regions of FoxO molecules.

## Materials and methods

### Sequence Data Collection

The DNA sequences and amino acids sequences of FoxO genes were downloaded from NCBI's GenBank . PSI-BLAST searches were conducted against the non-redundant database of vertebrate genomes at NCBI (*e*-value cutoff = 1e-24) using the amino acid sequences of Foxo1, Foxo3, Foxo4 and Foxo6 of mouse (gi: 56458, gi: 56484, gi: 54601 and gi: 329934) as queries. Only full length coding sequences were included in our analysis. Jalview 2.3 [31] was used to remove the sequences with the identity higher than 95%. A table with species names, abbreviations and accession numbers are provided in supplementary materials (Additional file 1).

### Sequence alignment and phylogenetic analysis

The sequences of FoxO proteins were aligned by MUSCLE [32] and the resulting alignment was manually optimized by BioEdit [33]. Incomplete sequences, and highly divergent regions or gaps resulting in uncertain alignments were excluded from the further analysis. The final data set included a total of 66 sequences from 19 species. The amino acid alignment was subsequently transformed into an aligned cds fasta file using PAL2NAL [34] which is a program to construct multiple codon alignments from matching amino acid sequences. The nucleotide alignment was then converted to nexus format with DnaSP [35] version 4.10 for phylogenetic analysis.

The full alignment of 66 sequences was used to perform the phylogenetic analysis. Tree reconstructions were done by the Bayesian method from the DNA alignment done in the MrBayes version 3.1.2 [36,37] software package, and rooted with the BfFoxO, Cifoxo and SpFoxO from amphioxus (Branchiostoma floridae), Ciona intestinalis and Strongylocentrotus purpuratus. We analyzed four independent runs, each using the general time reversible (GTR) model plus gamma distribution plus invariant sites model of molecular evolution (GTR+G+I), as determined by Modeltest version 3.7 [38]. We ran 2 million generation Markov Chain Monte Carlo simulations with four separate chains (three heated, one cold), with the first 500,000 generations discarded as burn-in. Trees were summarized for each independent run and compared to check for concordant topologies. The consensus tree of all compatible groupings among all runs was used in all analyses.

### Synonymous codon usage analyses

Codon usage bias was estimated by the effective number of codons (ENC; [39]), the frequency of optimal codons (*F*_*OP*_; [40]) and proportion of G and C in the third codon position (G/C 3rd). For ENC, lower values indicate stronger synonymous codon usage bias, while for *F*_*OP *_higher values indicate stronger bias. These measures were calculated for all genes using the CodonW program  and used to test whether the degree of synonymous codon usage biases in individual genes.

### Relative rate tests

The substitution rates of the FoxO genes were compared between different paralogous genes that had undergone duplication events recently, using the RRTree software [41]. The orthologs FoxOs (Cifoxo, BfFoxOA and SpFoxOl) from amphioxus (Branchiostoma floridae), Ciona intestinalis and Strongylocentrotus purpuratus were used as an outgroup. The null hypothesis is that the rate of substitution of the tested clade is the same as that of the reference group.

### Estimation of substitution rates and testing natural selection

We estimated the selective pressures acting on coding regions by applying a phylogenetic-based Maximum Likelihood (ML) analysis. ML estimated of the relevant parameters -as branch lengths and the ratio of the nonsynonymous (*d*_*N*_) to synonymous substitution rates (*d*_*S*_), ω = *d*_*N*_/*d*_*S*_-that were obtained using the *codeml *program implemented in the PAML package version 4 [42]. The ω parameter was used as a measure of the protein selective constraints [43]. These analyses were conducted under different competing evolutionary hypothesis. We first investigated whether the distribution of selective constraints acting on the each gene fluctuated across lineages; for that, we compared the fit to the data of the "one ratio" model (M0), which assumes a constant selective pressure across branches, with the "free ratios" model (FR), where the rate parameters were estimated independently in each lineage. We also examined other evolutionary scenarios; i) to determine which FoxO lineage had evolved at a different rate, as compared to the rest of the phylogeny, we applied a branch-specific model to the data. Sequences were divided into four groups according to their phylogenetic analysis, and each FoxO lineage was set as the foreground branch. ii) to detect sites under positive selection in four lineages, we applied three codon-based ML substitution models that are site-specific (i.e., models that allow variation in the ω ratio across sites) of [44] but assume the same selection pattern for a site in all lineages; iii) to investigate the existence of sites evolving under positive selection in only a specific lineage, we applied the modified branch-site model A of [45] in two consecutive tests (test1 and test2 in [46]) to the same alignment used for the site-based models. The model allowing for positive selection is denoted model A and the lineage to be tested is the foreground lineage, whereas the remaining ones are the background lineages; the multiple hypothesis testing problem [47] was taking into account using Bonferroni's correction [48]. The likelihood Ratio Test (LRT) was used to compare the fit to the data of two nested models, assuming that twice the log likelihood difference between the two models (2ΔL) follows a χ^2 ^distribution with a number of degrees of freedom equal to the difference in the number of free parameters [49].

We used the TreeSAAP version 3.2 [50] to determine the FoxO physicochemical properties affected by natural selection. This program for examining the effects of nonsynonymous substitutions on protein evolution compares the observed distribution of physicochemical changes inferred from a phylogenetic tree with an expected distribution based on the assumption of completely random amino acid replacement expected under the condition of selective neutrality. For all possible pairwise amino acid changes, the range of effect size for each of the 31 properties was determined and equally divided into 8 magnitude categories. Categories 1 to 3 indicate small variation in the amino acid characteristics while categories 6 to 8 represent the most radical substitutions. For all properties that differed significantly from neutrality, *Z-scores *were then calculated in each magnitude category to determine which classes contributed to this deviation. The critical *Z-score *values for *P *= 0.001 are 3.09, indicating positive selection on that magnitude category, and -3.09, which indicates negative (purifying) selection. That is, positive and negative *Z-scores *indicate positive and purifying selection, respectively. Radical substitutions affecting a particular property that occurred more frequently than expected by chance constituted the signature of adaptive evolution [51].

### Testing functional divergence and structure analysis

To study the functional divergence and structural differences after the gene duplication, we used the Diverge 2.0 software to estimate the type I (*θ*_I_) and type II (*θ*_II_) functional divergence coefficients [52,53] among paralogous proteins. Type I and type II refer to shifts in the evolutionary rate pattern after the emergence of a new phylogenetic cluster (indicative of changes in functional constrains), and amino acid replacements completely fixed between duplicates (resulting in cluster-specific alterations of amino acid physiochemical properties), respectively.

Genes which have been predicted to subject to positive selection were used to search for homologous sequences in the PDB database of protein structures  using Blastp [54,55]. The Rasmol  was used for all structural manipulations and highlighting the relevant amino acid replacements identified in the evolutionary analyses..

## Results

### Sequence similarity searches and multiple alignments

Available FoxO1, FoxO3, FoxO4 and FoxO6 sequences were retrieved from 19 species ranging from amphioxus (Branchiostoma floridae) to mammals. Additional file 1 outlines the sequences (protein and DNA) used in the phylogenetic analyses. The highly conserved forkhead domain remained in all alignments. It should be noted that additional FoxO genes for eutherians and teleosts were identified. Inclusion of these did not improve the reliability of the phylogeny, and as the aim of this study was to determine the evolutionary history of the FoxO gene family, only representatives from the major vertebrate clades were included.

### Phylogenetic analyses of FoxO gene lineages

**Figure 1:**
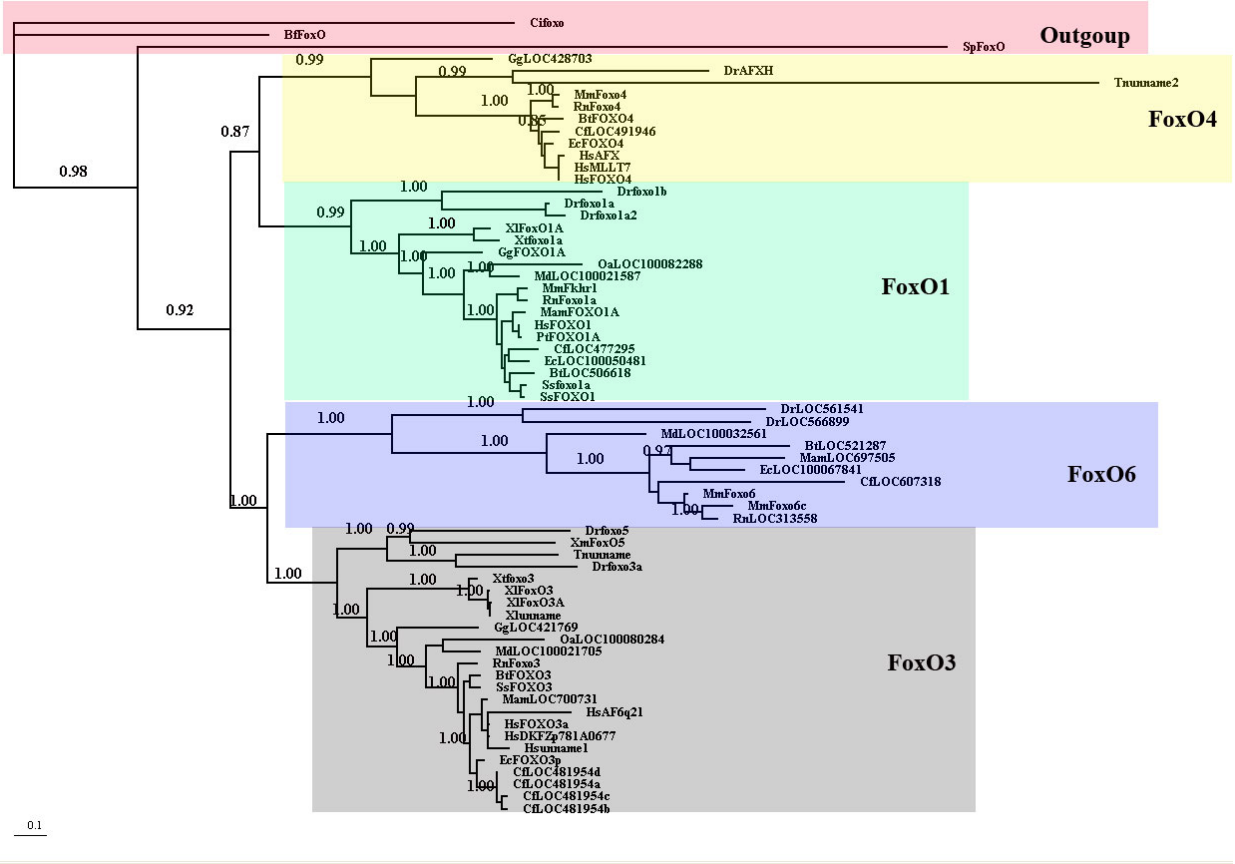
**Phylogenetic relationships of DNA sequences within the FoxO family**. Phylogenetic tree based on the nucleotide sequence data. The numbers indicate the Bayesian probabilities for each phylogenetic clade. Shaded boxes denote the four lineages and one outgroup. The scale bars represent codon substitutions per site.

To study the molecular evolution of vertebrate FoxO genes, we carried out phylogenetic inference analyses based on codon alignment and inferred their evolutionary history using Bayesian methods. We used the Bayesian posterior probabilities (*PPs*) of each node to evaluate clades support. Figure 1 shows the consensus phylogeny obtained for FoxO gene sequences. The vertebrate FoxOs were assorted well to four lineages according to their FoxO classification, all with high *PP *support values (a poorly supported position: 0.99 *PP*) indicating that the formation of the paralogous lineages occurred before the divergence of individual species, and the orthologs FoxOs (Cifoxo, BfFoxOA and SpFoxOl) from amphioxus (Branchiostoma floridae), Ciona intestinalis and Strongylocentrotus purpuratus were just located as an outgroup of their assigned lineages. From Figure 1, we inferred that two major duplications had occurred early in the vertebrate lineages. The first duplication led to the emergence of two lineages which evolved into FoxO3/6 and FoxO1/4, and the second duplication, also early in vertebrate evolution, resulted in FoxO6 and FoxO3, and FoxO1 and FoxO4. Phylogenetic tree shows that the FoxO6 gene cluster has long branches, an indication of fast-evolving lineage with a large number of structural changes accumulating on them.

### Synonymous codon usage analyses

**Figure 2:**
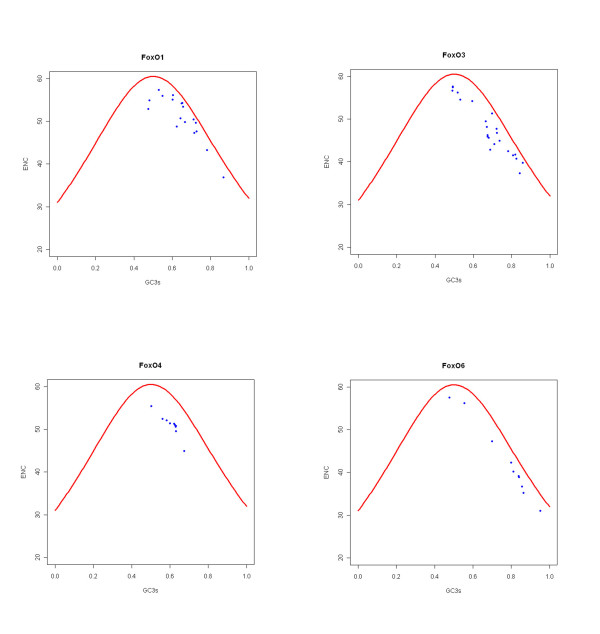
**The effective number of codons (Nc) plotted for each FoxO genes**. The FoxO genes highlighted in blue dot. The GC(ref) line -- shown in red -- is the expected position of genes whose codon usage is only determined by the GC content at the third positions of codons (GC3s).

We investigated the relationship between nucleotide content and codon usage by calculating different indices (Table 1) for each of the FoxO genes. We could see from Table 1 that the effective number of codons (ENC) decreased with the corresponding increase of GC3. The effective number of codons [39] is a measure of the evenness of codon usage among the 61 sense codons. An extreme case is that all codons are used equally frequently (given the observed frequencies of amino acids), then the effective number of codons is 61. Reversely, only single codon is used for each amino acid, the effective number of codons is reduced to 20. Therefore, FoxO6 gene was more biased than other FoxO genes as evidenced by their lower ENC values. In most cases, the observed number fell somewhere between the two extremes. Figure 2 shows the relationship between the effective number of codons (ENC) and the GC content at the third position of each gene (GC3). This Figure also contains a reference line (GCref) showing the expected position of genes whose codon usage is constrained solely by the nucleotide composition at the third codon position. From Figure 2, it can be seen that the observed value of ENC tracks the reference line quite closely. This indicates that the nucleotide composition at the third codon position is a major determinant of the effective number of codons.

**Table 1:** Mean values of GC%, GC3%, ENC, CAI and Fop of the FoxO genes

**Gene**	**GC%**	**GC3%**	**ENC**	**CAI**	**Fop**
FoxO1	0.5775 ± 0.0416	0.6478 ± 0.1009	51.0256 ± 5.1246	0.0661 ± 0.0119	0.3506 ± 0.0259
FoxO3	0.5797 ± 0.0538	0.6744 ± 0.1194	47.9308 ± 6.3169	0.0765 ± 0.0171	0.3851 ± 0.0283
FoxO4	0.5974 ± 0.0430	0.6082 ± 0.0459	50.9273 ± 2.5006	0.0660 ± 0.0129	0.3522 ± 0.0385
FoxO6	0.6773 ± 0.0867	0.7695 ± 0.1486	42.4620 ± 8.7015	0.0462 ± 0.0212	0.3032 ± 0.0599

Note: Mean ± Standard deviation

### Relative rates of evolution of FoxO6 lineage

Using the orthologs FoxOs (Cifoxo, BfFoxOA and SpFoxOl) from amphioxus (Branchiostoma floridae), Ciona intestinalis and Strongylocentrotus purpuratus as an outgroup, we evaluated the relative rates between FoxO gene clusters. The analysis (Table 2) revealed that the FoxO6 lineage exhibited accelerated nonsynonymous substitutions with respect to FoxO3 (*p-*value = 0.00163, Bonferroni correction) and FoxO1 (*p*-value = 0.0193, Bonferroni correction), and that FoxO4 genes were not accelerated with respect to the other FoxO lineages. Therefore, evolutionary-rate changes may have occurred following FoxO gene duplications in the evolutionary process.

**Table 2:** Evolutionary Rate of the FoxO Gene Families

**Lineage1**	**Lineage2**	**Ka1**	**Ka2**	**dKa**	**sd_dKa**	**ratio_Ka**	**P_Ka**
FoxO6	FoxO4	1.03324	0.997808	0.035436	0.046871	0.756029	0.449673
FoxO6	FoxO3	1.03379	0.902464	0.131326	0.041694	3.14977	0.001638
FoxO6	FoxO1	1.05943	0.952932	0.106501	0.04553	2.33915	0.019336
FoxO4	FoxO3	0.984938	0.921849	0.063089	0.044978	1.40267	0.16075
FoxO4	FoxO1	0.997224	0.942435	0.054789	0.048375	1.13257	0.257401
FoxO3	FoxO1	0.94013	0.949027	-0.0089	0.040392	-0.22028	0.825657

Note: Ka corresponds to the mean evolutionary rate measured as the number of nonsynonymous substitutions per site. dKa is the mean rate difference between the two lineages. sd_dKa is the standard deviation and ratio_Ka the ratio between dKa and sd_dKa. The P_Ka column corresponds to the *P *value associated to the test

### Selective constraints and functional divergence

**Figure 3:**
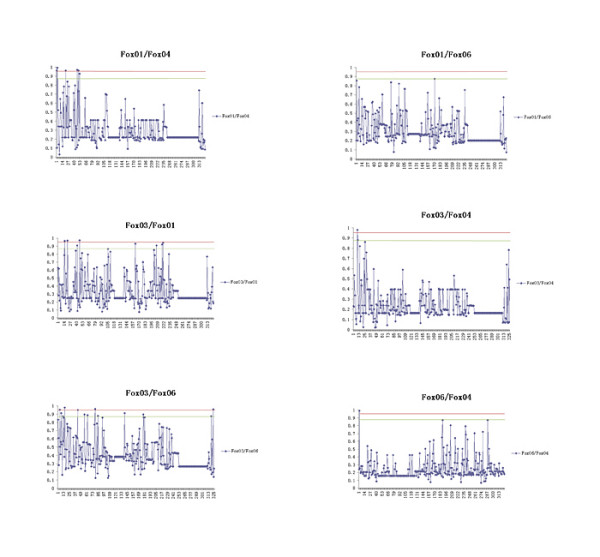
**Type I functional divergence among the FoxO members**. Posterior probability (*PP*) profiles of the site-specific type I functional divergence. The positions with gaps involved in each paralogous comparison were not considered. Red line indicates cutoff = 0.95, while green cutoff = 0.87.

Gene duplication-specific changes in the substitution rates (type I functional divergence) might reflect the difference in evolutionary rate at amino acid sites after gene duplication [52,53]. We found significant evidence of type I functional divergence for comparisons between different gene clusters (*θ*_*I *_= 0.23 ~0.40, *P *< 0.01; Table 3); namely, there were some amino acid sites with discrepancies in their evolutionary rate between these paralogous pairs. As expected, most amino acids had very low posterior probability (*PP*) values and, therefore, they would not be involved in the hypothetical functional divergence (Figure 3). Specifically, we detected 32 and 15 amino acid positions which presumably submitted to altered functional constraints when the *PP *threshold values were set to 0.87 and 0.95, respectively. Type I sites are defined as those with an amino acid that is conserved in one cluster but variable in the sister cluster, implying that the site is under structural/functional constraints in the first cluster that is absent in the variable cluster [56].

**Table 3:** Maximum likelihood estimates of the coefficient of functional divergence (θ) from pairwise comparisons between FoxO groups

**Comparison**	***θ*^a^**	**SE^b^(*θ*)**	**LRT^c^(*θ*)**	**sig**.
FoxO1 Vs FoxO3	0.33	0.05	50.89	P < 0.01
FoxO1 Vs FoxO4	0.29	0.04	41.23	P < 0.01
FoxO1 Vs FoxO6	0.3	0.06	20.43	P < 0.01
FoxO3 Vs FoxO4	0.23	0.05	19.86	P < 0.01
FoxO3 Vs FoxO6	0.4	0.05	57.92	P < 0.01
FoxO4 Vs FoxO6	0.24	0.05	21.61	P < 0.01

Note: ^a ^*θ *is the coefficient of functional divergence;

^b^SE(*θ*) standard error;

^c ^LRT(*θ*) is a likelihood ratio test;

Recently, a method has been developed to test for type II functional divergence [57]. Type II sites are those that are highly conserved in both clusters but are fixed for amino acids with different biochemical properties between sister clusters, implying these residues are responsible for the functional differences between these groups. Although at least one site with evidence of type II divergence was found for comparisons between FoxO1/FoxO3, FoxO3/FoxO4, and FoxO1/FoxO4 clusters, the *θ*_*II *_values are extremely small (*θ*_*II *_= 0.005 ~0.074) that highlighted the conservation between different clusters. These results are not unexpected given that this method calculates *θ *across all sites in an alignment and thus effectively averages site-wise *θ *values. With only ~3% of sites/cluster showing a pattern of type II divergence in our concatenated alignment, it is not likely that the ~9 possible type II sites have *θ*_*II *_values high enough to compensate for the extremely low *θ*_*II *_values of the over 300 sites with *θ*_*II *_effectively equal to zero. Our results are similar to the analysis of Hox-gene [30].

The analysis of the nonsynonymous to synonymous substitution rate ratio can also be used to detect functional differentiation. We estimated ω as an average over all sites and branches from the FoxO paralogus MSA and the ratio was substantially smaller than 1 (one ratio model ω = 0.084, Table 4) that indicated that purifying selection had been the predominant force acting on the evolution of these vertebrate FoxOs. Omega estimates for FoxO1, FoxO3, FoxO4 and FoxO6 phylogenies were 0.09583, 0.08311, 0.14088 and 0.13464, respectively. Selective constrains, however, are unevenly distributed across the phylogeny (FR model; 2ΔL = 421.20, *P *< 0.001). We then ran the branch model using each FoxO lineage as the foreground branch. In this model the estimated ω_1 _was 0.0758 for the FoxO1, and 0.0898 for the background branches. A LR test indicated that the two-ratio model was not significantly different from the M0 model (2ΔL = 3.05, *P *> 0.05, df = 1, Table 4). In contrast to FoxO1 analysis, the ω values of the FoxO3, FoxO4 and FoxO6 lineages were different from the rest of the phylogeny as the LR tests indicated that the two-ratio model fit the data better than the M0 model for these three genes (*P *< 0.05). Unfortunately, the ω estimates for FoxO3, FoxO4 and FoxO6 were not indicative of positive selection, they were rather indicative of relaxed constraint.

**Table 4:** LRTs done to detect heterogeneous selection regimes among lineages for each gene

**model**	**df**	**Parameter estimates**	**lnL**	**2⊿l**	**p value**
Branch-specific models			-22943.8		
M0(one-ratio)		ω = 0.08442			
FoxO1					
two-ratio vs one-ratio	1	ω_0 _= 0.0898ω_1 _= 0.0758	-22942.3	3.047108	p > 0.05
FoxO3					
two-ratio vs one-ratio	1	ω_0 _= 0.0910ω_1 _= 0.0730	-22941.3	4.87108	p < 0.05
FoxO4					
two-ratio vs one-ratio	1	ω_0 _= 0.0811ω_1 _= 0.1044	-22941.8	3.983174	P < 0.05
FoxO6					
two-ratio vs one-ratio	1	ω_0 _= 0.0786ω_1 _= 0.1358	-22935.8	16.00121	P < 0.01

Along with lineage heterogeneity, variations in ω across sites can also occur. Theoretically, different protein regions with different functions may experience different selection pressures, which can be tested by fitting the data to a model comprising different site classes. The results were shown in Table 5, for each lineage, the M3 vs M0 LRT was significant, indicating that one category of ω wasn't fit data well to describe the variability in selection pressure across amino acid sites. The tests contrasting the models M1a against M2a resulted in the *P *value of 1 for all the groups suggesting a lack of power and the amino acid changes within each cluster were neutral or under negative selection. M1a, the parameter estimates for the least parameter rich model describes that most sites with low ω estimates (indicative of strong selective constraints), that is, 82% of FoxO1 sites were under strong purifying selection, compared to 83% for FoxO3, 74% for FoxO4 and 66% for FoxO6. The test using M7 and M8, which allows for beta-distributed site-specific ω ratio, detected 2 groups under possible positive selection at 0.05 significance level, one with ω = 1.36 and the other with ω = 127.02 (Table 5). In order to test whether the estimated ω is significantly greater than 1, model M8 was compared with a more restricted null model (M8a). For FoxO3 gene, Model M8 did not significantly differ from model M8a (2ΔL = 0.067, *P *> 0.05, df = 1). For FoxO6 gene, ω = 127.02 was significantly different than 1 (2ΔL = 51.92, *P *< 0.01, df = 1). We also used the BEB estimation method in model M8 [46] to identify sites under possible positive selection.

**Table 5:** Site model analyses for the FoxO1, FoxO3, FoxO4 and FoxO6 phylogenies

	**Models comparison**							
				
	**M3 vs M0**		**M2a vs M1a**		**M8 vs M7**			
Gene	2ΔL = (L1-L0)	p-value	2ΔL = (L1-L0)	p-value	2ΔL = (L1-L0)	p-value	ω- value	Positively selected sites
FoxO1	580.7848	p < 0.01	0	1	0	1		
FoxO3	902.91	p < 0.01	0	1	8.23	p < 0.05	ω = 1.36	66 L (p > 0.90)
FoxO4	178.24	p < 0.01	0	1	0	1		
FoxO6	455.31	p < 0.01	0	1	69.66	p < 0.01	ω = 127.02	264K* 266P* 434G* 439T*

Note: *P > 0.95

Since positive selection will likely affect a few amino acids at specific lineages on the phylogeny, models estimating ω ratios averaged by codons or by lineages are certainly highly conservative. For this reason, a branch-sites method has been developed that allows variation in ω across individual codons on a specific lineage [46,58]. This model (MA) designates two categories of branches, again foreground and background, where positive selection is modeled only on the foreground branch. We then applied the branch-site approach (using some pre-specified branches, i.e., foreground branches), designating each FoxO gene as the foreground branch, to assess whether molecular adaptation occurred in the evolution of the FoxO genes. The results of this analysis exhibited several positions with evidence of relaxed selection (the test 1 was significant) (Table 6). However, we could not reject the null hypothesis of the test 2 (ω_2 _= 1) (result not shown); thus, these analyses do not provide any evidence for directional selection on the FoxO lineages.

**Table 6:** Parameter estimations and likelihood ratio tests for the branch-site models

	**df**	**Parameter estimates^a^**	**lnL^b^**	**2⊿^c^**	**P value**	**Positive selected sites**
FoxO1						
MA Vs M1a (test 1)	2	p_0 _= 0.70592 p_1 _= 0.18493 (p_2 _= 0.10915) w_0 _= 0.07205 (w_1 _= 1.00000) w_2 _= 1.00000	-22667.231	81.89419	P < 0.01	213S** 216S* 219S* 252M* 276V** 285P* 296L** 306A** 340F* 360E*
FoxO3						
MA Vs M1a (test 1)	2	p0 = 0.77299 p1 = 0.10453 (p2 = 0.12248) w0 = 0.06958 (w1 = 1.00000) w2 = 1.00000	-22646.766	122.8257	P < 0.01	5H** 25D* 26F** 33D** 34L**37N** 217A* 231G** 329G*
FoxO4						
MA Vs M1a (test 1)	2	p0 = 0.67705 p1 = 0.18055 (p2 = 0.1424) w0 = 0.07468 (w1 = 1.00000) w2 = 1.00000	-22660.108	96.14052	P < 0.01	7V** 173R** 194T** 201I** **202**L** 211F**223H* 225P** 242T* 254R** 314S**
FoxO6						
MA Vs M1a (test 1)	2	p0 = 0.58516 p1 = 0.13030 (p2 = 0.28454) w0 = 0.06572 (w1 = 1.00000) w2 = 1.05311	-22527.632	361.0936	P < 0.01	42Q** 46K** 155I* 164T** 165N** 173R* 174E** 176E** 178L** 179F** 180C** 188I* 189V** 203L* 207R* 223H** 230I** 231G* 232Y** 233K** 234N** 237Y** 258S** 265N* 269T** 271E** 272N** 273E** 274V** 275H** 276V** 277S** 278Q* 279G** 280L** 281H** 282P** 283S** 286N* 314S** 316V** 320H** 330Y* 366T** 367G** 368T** 369P*

Note: ^a^The number of free parameters;

^b^Likelihood of the model;

^c^2(l_1_-l_0_);

* P > 0.95;

** P > 0.99;

The bold amino acid residues were also found to be implicated in the functional divergence (implemented in Diverge 2.0) between FoxO

The molecular adaptation processes occurred after the gene duplication event were also investigated by comparing the magnitude of the physicochemical changes produced by the observed amino acid replacements with those expected at random [59]. We used the program Tree-SAAP [50] to model how 31 different physicochemical properties were affected by amino acid substitutions in each FoxO gene. Consistent overrepresentation of radical amino acid changes (i.e., categories 7 and 8) would suggest repeated adaptive substitution [51]. The results indicated that, some amino acid replacements altering these physicochemical properties in the FoxO1 and FoxO3 proteins accumulated more (or less) often than expected by chance (likely reflecting fitness differences) (supplementary materials (Additional file 1)). Moreover, for each physicochemical property, the distribution of the *Z-scores *across 8 magnitude classes [51] indicated that, amino acid substitutions occurred less often than expected by chance at the most extreme magnitude-classes (supplementary Additional file 1); these FoxO1 and FoxO3 protein properties, therefore, were likely evolving under purifying selection. For FoxO4 and FoxO6 genes, less physicochemical properties were affected by amino acid substitutions. The FoxO6 gene, on the contrary, seems to evolve positive selection, because category 8 occurs more frequently than expected by chance for 2 of the properties (alpha-helical tendencies and compressibility).

### Spatial distributions of possible selected FoxO6 Sites on three-dimensional structure

**Figure 4:**
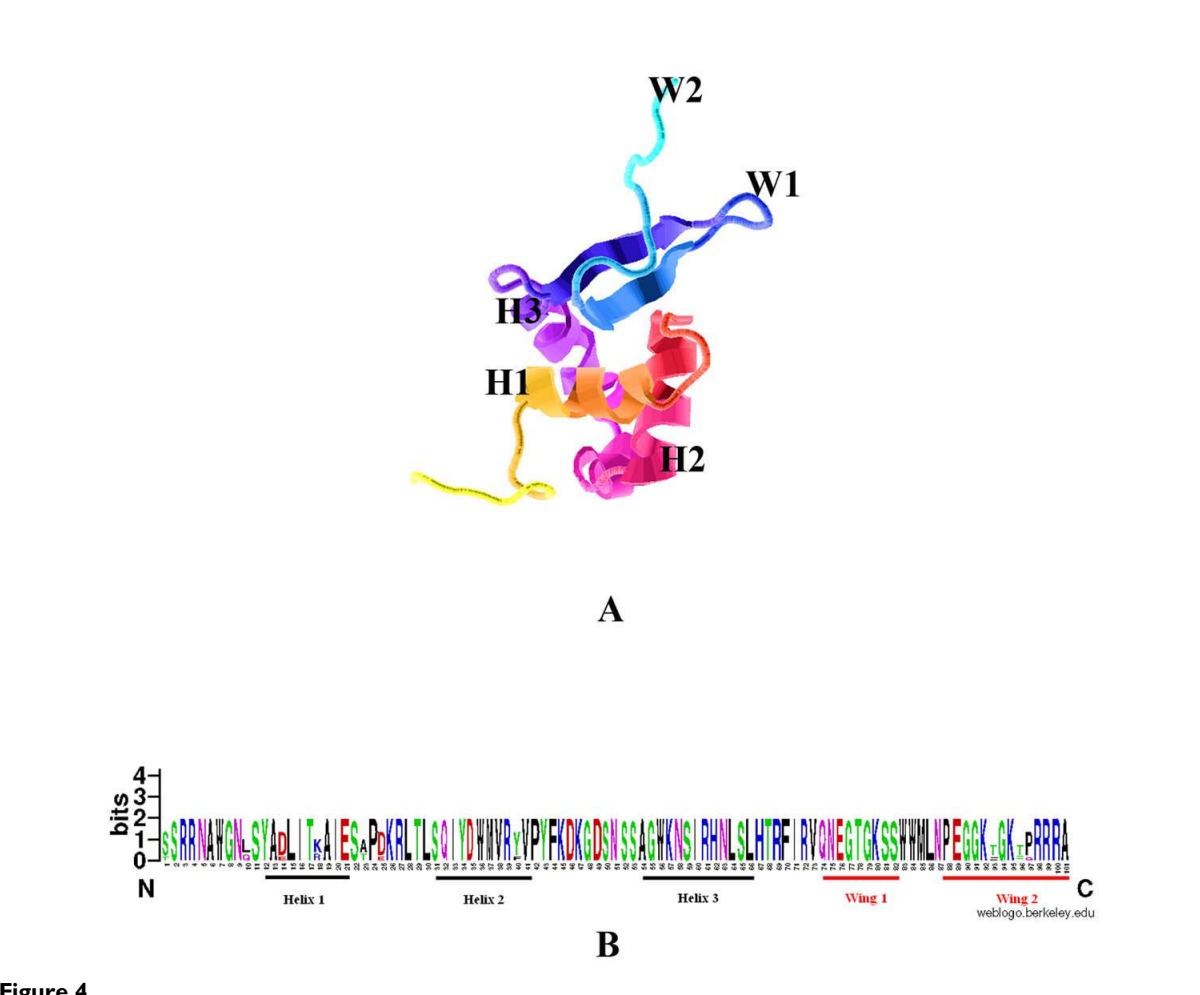
**The modeled structure of mouse Foxo6**. A. The structure of the forkhead domain; B. Sequence logo of the forkhead domain and surrounding amino acids.

Because of the evidence for possible positive selection on FoxO6, we predicted positively selected codon sites using a Bayes empirical Bayes (BEB) method [45]. The sites under selection in FoxO6 are listed in Table 5. Four codon sites were identified as positively selected at a BEB posterior probability threshold of 95%. Moreover, 7 amino acid residues presumably submitted to altered functional constraints were identified by both PAML 4 and Diverge 2.0 analysis (Table 6). In order to plot positive selected sites onto mouse (Foxo6) three-dimensional model, we first built an energy-minimized model using a homology modeling approach [60]. The PDB entry with the highest sequence similarity -identified in the PSI-BLAST- corresponds to the human FOXO3A (PDB: 2k86). We used this entry as a template for the modelling. The *in silico *stereochemical quality analysis [61] indicated that the generated model had a moderate quality (with the percentage of residues in most favored regions being no lower than the 82.8%), with only 1.1% in disallowed regions. As expected, the modeled structure was roughly similar to the template, with the three helices and two wing loops typical of the Fox family in equivalent positions and with a similar predicted folding (Figure 4). Taken together, these data suggested that the model was stereochemically valid, and therefore suitable for further sequence-structural analysis. Unfortunately, we could not map any positive selected sites onto the surface of the 3D structure (Figure 4A) because the crystal structures about Fox proteins are mainly focused on the forkhead DNA-binding domain. Whereas the positive selected sites were mainly located in the region of N-terminal and C-terminal of FoxO6, which also indicated that FoxO6 underwent strong constraint on the forkhead domain as well (Sequence logo of the forkhead domain, Figure 4).

## Discussion

It has long been know that FoxO transcription factors play important roles in regulating various signals, which translate various environmental stimuli into dynamic gene expression programs to influence many physiological and pathological processes, including cancer and aging. The functions of FoxO proteins are regulated at multiple levels, which include but are not limited to phosphorylation, ubiquitylation and acetylation. Interestingly, all of these activities affect nuclear/cytoplasmic trafficking of FoxO proteins. The specific function of each member of this family is different [14]. As the accumulation of gene sequences in the database, it is feasible to explore the functional diversity from a phylogenetic perspective. We performed firstly to the resolution of the evolutionary relationships of these FoxOs using molecular sequence data. Whereas, the incorrect phylogenetic topology resulting from mutationally saturated positions, inadequate modeling of the evolutionary process and systematic bias due to variable rates of evolution among species or within sequences [62] may make LRT generate many false positives. Anisimova et al (2003) examined the effect of assuming a "wrong" tree [63], and he found that LRT falsely suggested positive selection in 96% of the replicates in the M0-M3 comparison and in 86% of the replicates in the M7-M8 comparison at the ΰ = 5% significance level. In order to overcome this problem, we adopted a number of ways in combination. Firstly, the addition of more taxa to the dataset: denser sampling of species can reduce the effect of long branch attraction (LBA) by reducing the overall distances between taxa. Secondly, we used the best model of DNA substitution, determined by Modeltest version 3.7 [38]. And finally, our inclusion of enough sequences in each lineage helped alleviate loss of LRT power from short conserved sequences. From phylogenetic result, we focused on the 2 main duplications along the evolutionary history of FoxO genes, the FoxO1-FoxO4 and the FoxO3-FoxO6 duplication, which formed four gene lineages (all with the high confidence values, nearly 100% posterior probability in Bayesian analysis) and used for further analysis.

Codon bias is largely thought to be due to weak selection acting to optimize protein production [64-66]. Selection intensity for codon usage bias, therefore, is expected to vary among genes. Our survey of synonymous codon usage in FoxO genes revealed a strong and consistent pattern of codon bias in genes with FoxO6 relative to those with FoxO1, FoxO3 and FoxO4 (Table 1). At the same time, there appears to be some conflicting results observed between *F*_OP _and ENC, which may be caused by differences in the way that the two methods estimate codon bias. *F*_OP _is based on the frequency of a set of species specific "optimal" codons, while ENC is based on the observed number of codons used for each amino acid. Thus it is possible for the two methods to give different estimates of codon bias.

It is widely accepted that gene duplication can create opportunities for functional divergence in paralogues. Divergence is thought to occur where one duplicate retains the original protein function and the other accumulates changes, (either through redundancy or by positive selection) or alternatively, through the partitioning of the functions of an unduplicated ancestor protein. Whatever the mechanism, if functional divergence has occurred between duplicated genes, then it should be observable as changes within their coding regions.

The functional divergence of FoxO genes has been studied by [14]. The branch length leading to the FoxO6 clade is extended relative to other FoxO genes in gene phylogeny, (Figure 1). This suggested that after the duplications, FoxO6 evolved at a faster rate than other FoxO genes. This result was confirmed by significant relative rate test results for FoxO gene lineages (Table 2). In this sense, we performed type I functional divergence analysis, and we detected significant type I divergence among FoxOs. The comparison between the FoxO3 and FoxO6 groups showed the highest value for *θ *(0.40 ± 0.05), suggesting that these two groups had diverged considerably more at the functional level. Next, DIVERGE was used to establish the posterior probability of type I divergence at each site in the alignment, employing two cut-off posterior probability values of 0.87 and 0.95. However, the cutoff value for residue selection is an empirical decision and is expected to depend on the intrinsic properties of the protein family being analyzed. Thus, we predicted 32 candidate functional divergence-related sites using 0.87 as a cutoff value (supplementary materials (Additional file 1)). When we narrowed our criteria to 0.95, we got 15 candidate residues as the most likely candidate sites for type I functional divergence (supplementary materials (Additional file 1)), but we lacked a way to verify how the rate-shift in these sites contributed to functional divergence among the FoxO gene groups. For comparative purposes, the same alignment and phylogeny was submitted to a ML LRT, which, like the Bayesian method provided a statistical framework where evolutionary rate shifts at particular protein positions could be established [46]. At last, the statistically most likely positions predicted to underlie functional divergence were agreement by both methods, particularly for the highest-ranking candidates (Table 6).

In this study we used codon substitution models implemented through a maximum likelihood framework to estimate the rate of evolution at silent and replacement sites in FoxO1 and its paralogs, FoxO3, FoxO4 and FoxO6. Different models were used to investigate variation in the rate of evolution between lineages of a phylogeny, and to estimate ω for specific lineages and sites across phylogenies. Our objective was to determine the mode of evolution on each FoxO gene lineage, and to determine whether increased positive selection or decreased constraint led to the functional divergence of FoxO genes. As we have demonstrated, variation among branch and sites was observed in the FoxO6 phylogeny. Moreover, physicochemical amino acid properties analysis also provided evidence that the entire FoxO6 gene had experienced repeated episodes of adaptive evolution. The site models showed that adaptation had appeared at four sites located at C-terminal of FoxO6. For FoxO3 gene, Model M8 fits data significantly better than Model M7 (2ΔL = 8.23, df = 2, *P *< 0.05), and because 1.5% of sites are located in the positively selected site class with ω = 1.36, weak positive selection may be indicated with this comparison. However, it has been found that a poor fit of the data to a beta distribution may result in a high frequency of significant tests when comparing models M7 and M8 even in the absence of positive selection. To take account for the elevated type I error rates, the original model M8 was compared with a more restricted null model (M8a), where the extra site class was constrained to have ω = 1. When performing this analysis with the sequence data from the FoxO3 gene, model M8 did not significantly differ from model M8a (2ΔL = 0.067, df = 1, *P *> 0.05), indicating that the estimated ω = 1.36 was not significantly different than 1 and that there was little indication of positive selection in this gene. Further, our observation of strong purifying selection being the primary mode of evolution throughout the FoxO phylogeny is consistent with the findings of a recent study about forkhead family [67,68].

When we performed branch-site model analysis, we found relaxed functional constraint was most consistent with the molecular evolutionary analyses of the FoxO data. Our conclusion is in contrast to a previous study which concluded that one site was found to be under positive selection in the FoxO3 lineage [67]. In our paper, we applied the modified branch-site model A of [45] in two consecutive tests (test1 and test2 in [46]) to the same alignment used for the site-based models, but we could not detected the positive selection site. To determine why these two articles are giving drastically different results, we had a look at the sequences used for branch-site model analysis in [67], and we found that only 12 sequences (4 for FoxO3) used for testing evolution selection. Test 2 is a more direct test for identifying positive selections in the foreground branch, and significant LRT from test 1 can be resulted from either positive selection or relaxed selective constraint in the foreground branch [46], however, the power of this method may be limited when sample size or divergence time is low [46]. Therefore, we concluded that the contradiction between our results and the previous study [67] due to the number of sequences used for analysis. Moreover, our work on site-model analysis, relative rate test and physicochemical changes indicated that FoxO6 was under positive selection. Four positive selected sites were identified by site-model analysis, two (264K, 266P, corresponding to mouse Gly^337 ^and Pro^339^) of them fell into the region of non-conserved optimal PKB motif in the C-terminal part (Thr^338^) [69]. The C-terminal PKB recognition sequence is not conserved in FoxO6 [13]. Besides a PKB phosphorylation motif, this region contains a stretch of 3 additional serine residues, present in the other members of the FoxO group, but FoxO6. All these suggest that these serines may be functionally important in the other sequences analyzed with the exception of FoxO6 gene, and positive selection may lead to functional divergence between FoxO6 and the other members too. Another two positive selective site are Gly^545 ^and Pro^550 ^in the mouse Foxo6, and the functional role remains elusive. That is to say that the real reason for their accelerated evolution is unclear. However, it should be mentioned that there is a gap in the knowledge of the relationship between amino acid sequence and structure for full-length FoxO sequence, and we are unable to speculate on the particular role of this region in these FoxO6 genes. Unfortunately, the shared evolutionary history and molecular selection alone cannot be used as the unique criterion to infer protein function, and the true nature of each FoxO gene needs to be determined experimentally and independently. Therefore, the positively selected site may play an important functional role and could represent an interesting target site for future mutagenesis experiment thus facilitating our understanding of the structure-function relationships in FoxO genes. Molecular testing is required to validate this hypothesis. The result from branch-site analysis (relaxation of functional constraints) of FoxO6 also differs somewhat from previously signature of positive selection. We infer that the weak positive selection and multiple branches are considered as foreground branch may explain this phenomenon, because power will be reduced unless the same sites and selective constraints are occurring along all foreground branches [46].

## Conclusion

Genomic data have provided an opportunity to gain a better understanding about the evolution of FoxOs using phylogenetic analyses. The FoxO gene family phylogeny showed that two duplications took place early in the evolution of vertebrates and triggered diversification of the FoxO gene family into four groups. However, further genome projects on a greater diversity of evolutionary lineages would help better understand the gene-duplication history. The relative rate analysis and physicochemical changes indicated that FoxO6 seemed to be different from other members. Evolutionary rate analysis showed that molecular adaptation can also play an important role in the evolution of this gene family. Indeed, positive selection was likely involved in the functional differentiation of FoxO6 gene; likewise, relaxed selection might play important roles over evolutionary time and shape variation of some members of the family. Considering the evolutionary history of the FoxO gene family, we provided insight into which amino acid residues might have undergone positive selection and could be targeted for site-directed mutagenesis. However, the identification of four sites under positive selection requires supporting evidence from further functional experiments to demonstrate the adaptive character of the amino acids. All these studies and experiments will certainly contribute to better understand the precise role of natural selection and functional divergence of this family.

## Authors' contributions

MH, Wang conceived and supervised all research. QS, Wang helped with the Type I analysis. XZ, Zhang and HB, Zhao gave the suggestion on the analysis of physicochemical properties. YC, Pan gave the suggestion on discussion and approved the final version.

## Supplementary Material

Additional file 1 **Excel spreadsheet containing**: A list of species, species abbreviations, and accession numbers for sequences used in the study/A list of statistically significant physicochemical amino acid properties for each FoxO gene/A list of the candidate residues as the most likely candidate sites for type I functional divergence. Click here for file
